# The Global Platform Economy: A New Offshoring Institution Enabling Emerging-Economy Microproviders

**DOI:** 10.1177/0149206318786781

**Published:** 2018-08-23

**Authors:** Vili Lehdonvirta, Otto Kässi, Isis Hjorth, Helena Barnard, Mark Graham

**Affiliations:** University of Oxford; University of Pretoria; University of Oxford

**Keywords:** selection/staffing, developing countries, international management, transaction cost economics

## Abstract

Global online platforms match firms with service providers around the world, in services ranging from software development to copywriting and graphic design. Unlike in traditional offshore outsourcing, service providers are predominantly one-person microproviders located in emerging-economy countries not necessarily associated with offshoring and often disadvantaged by negative country images. How do these microproviders survive and thrive? We theorize global platforms through transaction cost economics (TCE), arguing that they are a new technology-enabled offshoring institution that emerges in response to cross-border information asymmetries that hitherto prevented microproviders from participating in offshoring markets. To explain how platforms achieve this, we adapt signaling theory to a TCE-based model and test our hypotheses by analyzing 6 months of transaction records from a leading platform. To help interpret the results and generalize them beyond a single platform, we introduce supplementary data from 107 face-to-face interviews with microproviders in Southeast Asia and Sub-Saharan Africa. Individuals choose microprovidership when it provides a better return on their skills and labor than employment at a local (offshoring) firm. The platform acts as a signaling environment that allows microproviders to inform foreign clients of their quality, with platform-generated signals being the most informative signaling type. Platform signaling disproportionately benefits emerging-economy providers, allowing them to partly overcome the effects of negative country images and thus diminishing the importance of home country institutions. Global platforms in other factor and product markets likely promote cross-border microbusiness through similar mechanisms.

From the 1990s onward, the adoption of new information and communication technologies has led to a gradual “tradability revolution” where it has become increasingly possible to relocate service production to offshore destinations (Doh, 2005; Dossani & Kenney, 2007). The main benefits that firms seek from service offshoring are reduced labor costs and access to skilled labor (Bunyaratavej, Doh, Hahn, Lewin, & Massini, 2011), and its main modes are outsourcing to an arm’s-length service provider and offshoring to a captive subsidiary (Bunyaratavej et al., 2011; Bunyaratavej, Hahn, & Doh, 2007). In recent years, a new offshoring mode appears to have emerged alongside these two established modes: one-person microproviders that serve clients around the world in transactions conducted via online platforms (Horton, 2010; Lin, Liu, & Viswanathan, 2016). The purpose of this article is to introduce this *global platform economy* and theorize it in the context of the international business (IB) literature on global sourcing.

A frequently used theoretical approach to explaining the choice between arm’s-length outsourcing and captive offshoring in the IB literature is transaction cost economics (TCE), which highlights the role of transaction costs and information asymmetries that cause cross-border markets to fail and lead firms to internalize transactions instead (Buckley & Casson, 2009; Chen, 2010; Williamson, 2008). To mitigate such cross-border information asymmetries and win clients, arm’s-length outsourcing providers frequently engage in costly reputation-building activities (Elitzur & Wensley, 1997; Levina & Ross, 2003; Oza, Hall, Rainer, & Grey, 2006). This is particularly important for firms from emerging-economy countries, which are further disadvantaged by negative country images associated with their countries of origin (Ramachandran & Pant, 2010). Partly because of the substantial resources required by such reputation-building activities, emerging-economy outsourcing providers are typically large firms or subsidiaries of foreign multinational enterprises (MNEs). Small providers struggle to build credibility with international clients and instead end up working as subcontractors or employees to larger providers (Foster, Graham, Mann, Waema, & Friederici, 2018).

However, the global platform economy appears to be challenging the dominance of large providers and MNEs. One-person microproviders are supplying services ranging from software development to copywriting and graphic design, and their business appears to be growing rapidly (Kässi & Lehdonvirta, 2016). They are located around the world, including in established offshoring destinations as well as in emerging-economy countries not previously associated with offshoring (Graham, Hjorth, & Lehdonvirta, 2017; Kuek et al., 2015). Their clients range from start-up firms to some of the world’s largest corporations and MNEs (Corporaal & Lehdonvirta, 2017; Kuek et al., 2015). For human resources managers, this implies that managing global work *for* an MNE may not always be managing global work *in* the MNE.

Given the information asymmetries and liabilities of origin inherent to cross-border trade, how do these microproviders survive and thrive? We adapt signaling theory (Connelly, Certo, Ireland, & Reutzel, 2011; Spence, 1974) to theorize the information problems that microproviders and their clients face and the role that global online platforms play in resolving them. We test our models’ predictions with 6 months of digital trace data from a leading online labor platform. We further integrate findings from semistructured interviews with microproviders in Southeast Asia and Sub-Saharan Africa to validate and augment the quantitative findings, a methodological innovation that mitigates some of the risks of working with “big data.”

This study contributes first to the literature on the fragmentation of the global value chain (Mudambi & Puck, 2016). This has been mainly theorized in the context of offshoring and outsourcing (S. Manning, Larsen, & Kannothra, 2017), but we extend the conversation to global labor platforms and theorize them as an institutional form that addresses cross-border information asymmetries that exclude small providers from global markets. Global platforms serve a similar function as country-level institutions that reduce information asymmetry, but they are global in scope and thus diminish the importance of the home country institutional context to providers’ international performance.

We also show the value of combining a TCE perspective with signaling theory to understand how microproviders, mainly from emerging economies, secure projects across national borders. Scholarship thus far has focused on larger firms, but we introduce the concept of microproviders. In examining the determinants of their performance, we show that a central challenge for these providers is to signal their competence and that the platform plays a key role in enabling them to signal their competence in various ways. Out of the different signaling options available, platform-generated signals are the strongest predictors of earnings. Because emerging-economy microproviders owe their market access to global platforms, they are highly dependent on them. This has implications for value capture, and we conclude with reflections for future areas of research.

## The Global Platform Economy

The so-called sharing economy or platform economy has become a prominent concept in scholarly and lay discussions in recent years (Hamari, Sjöklint, & Ukkonen, 2015; Mair & Reischauer, 2017; Sundararajan, 2016). While the two terms are often used near synonymously, *sharing economy* at least originally referred to amateur or noncommercial transactions, whereas *platform economy* also encompasses commercial transactions (Parker, Van Alstyne, & Choudary, 2016). No single authoritative definition of either term exists, but a universal characteristic of various definitions is that they place emphasis on individuals rather than organizations as the primary economic actors: “the supply of capital and labor comes from decentralized crowds of individuals rather than corporate or state aggregates” (Sundararajan, 2016: 27). Another near-universal characteristic is that these individual participants are organized by digital platforms that match suppliers and demanders as well as perform various management-type functions, such as quality control (Boudreau & Hagiu, 2009; Mair & Reischauer, 2017).

Discussion on the platform economy has focused on topics such as its potential to enable more efficient resource use (Belk, 2014; Botsman & Rogers, 2011), the threat it poses to incumbent firms and business models (Evans & Schmalensee, 2016; Parker et al., 2016), and the challenges it presents to standard employment (Davis, 2016; Prassl & Risak, 2016). Platforms are typically theorized through one of two main approaches. One approach is to model platforms as two- or multisided markets (Eisenmann, Parker, & Van Alstyne, 2006; Evans & Schmalensee, 2016). This approach is particularly useful for examining platform growth strategy and competition between platforms (Zhu & Iansiti, 2012). Another approach is a TCE approach, where platforms are seen as institutions whose efficient form is determined by technology and other exogenous factors. Already in 1987, Malone, Yates, and Benjamin posited that if technology drives down the cost of market transactions relative to internal firm transactions, then activities hitherto organized within firms will be externalized into markets. An early interpretation of platforms was that they are precisely such technology-enabled markets, breaking down hierarchies into free-floating one-person firms (Botsman & Rogers, 2011; Malone, 2004). More recent accounts have started to emphasize the influence that platforms’ internal mechanisms have on outcomes to the participants (Mair & Reischauer, 2017; Shevchuk & Strebkov, 2017). According to one recent view, platforms are “hybrid” institutions combining features of markets and hierarchies (Sundararajan, 2016). The TCE approach is particularly useful for explaining platforms’ emergence in a wider institutional field, such as an industry, and for understanding the incentive structures that they present to participants.

While most of the discussion has focused on platforms’ effects within high-income countries, there is also an important global dimension to the phenomenon. A subset of platforms, which we term *global platforms*, are focused on connecting supply and demand across the Internet-connected world (Ghani, Kerr, & Stanton, 2014; Horton, 2010; Kuek et al., 2015; Lehdonvirta, 2018), the greater part of which now consists of low- and middle-income countries (Graham, De Sabbata, & Zook, 2015). In particular, so-called online labor platforms (Horton, 2010) or online gig platforms (Kässi & Lehdonvirta, 2016) connect individual service providers with clients around the world to provide services as diverse as software development, graphic design, article writing, data entry, voice acting, and accounting.

Many of the service providers are located in emerging economies, especially in established business process offshoring destinations, such as India and the Philippines, and in information technology offshoring destinations, such as Eastern Europe, but also in other lower-income regions, including in Sub-Saharan Africa (Graham et al., 2017; Kässi & Lehdonvirta, 2016; Kuek et al., 2015). These providers are similar to the contract professionals of Osnowitz (2010) and the “hired guns” of Barley and Kunda (2006), but they include both highly skilled professionals and providers of routine administrative services. Moreover, they provide their services entirely remotely, typically for foreign clients who benefit from hyperlocal differences in the cost and availability of different skills across the globe (Corporaal & Lehdonvirta, 2017).

This global platform economy presents some notable differences to conventional modes of offshoring. One difference is that conventional offshore outsourcing providers are typically companies of significant size (S. Manning, 2013; S. Manning et al., 2017). For instance, in the Offshoring Research Network’s global survey, even “small” providers were defined as having up to 500 employees, and 25% of the providers surveyed had more than 10,000 employees (S. Manning, 2013). The idea that global providers could be one-person microproviders differs significantly from past empirical literature and challenges how human resources are managed across borders. Another novelty is the fact that transactions are mediated from start to finish by a third-party digital intermediary.

The global platform economy thus appears to represent a new type of offshoring institution, distinct in important ways from conventional offshoring modes. Its outward characterizing features could be summarized as *glocalization, platformization*, and *individualization.* By *glocalization*, we refer to the integration of local differences in the cost and availability of skills to a standardized global structure that enables their exploitation. The term is borrowed from cultural studies of globalization, where it is used to highlight “globalized forces” that structure local entities while maintaining and “valu[ing] diversity in them” (Gould & Grein, 2009: 238). By *platformization*, we refer to the introduction of the online platform as an active intermediary between supply and demand. By *individualization*, we refer to the puzzling elimination of returns to scale, resulting in a supplier base that consists of one-person microproviders. Next we lay out a theoretical context and craft a model of this institutional form that results in testable hypotheses.

## Theory

The IB literature recognizes a variety of costs, frictions, and information asymmetries that hinder cross-border trade (Kostova & Zaheer, 1999; Malhotra & Gaur, 2014; Zaheer, 1995). It also recognizes that emerging-economy firms face additional barriers to international market entry in the form of liabilities of origin (Pant & Ramachandran, 2012; Ramachandran & Pant, 2010). A significant achievement of IB literature has been to use TCE-based theorizing to explain different foreign market entry modes as institutional forms arising in response to such costs. The MNE arises when the costs of the arm’s-length market are so high that it is equally or more economical for the firm to internalize the transaction (Buckley & Casson 1976; Buckley & Casson, 2009; Chen, 2010; Hennaert 2009). Arm’s-length markets persist if firms can afford to take costly actions to overcome the barriers (Bell, Filatochev, & Rasheed, 2012; Kostova & Zaheer, 1999; Luo, Shenkar, & Nyaw, 2002; Mezias, 2002). These costs tend to exclude especially smaller firms in emerging economies from accessing international markets (Foster et al., 2018). A subset of the IB literature, global sourcing literature, uses analogous TCE-based theorizing to explain the emergence of the two main conventional offshoring modes: offshoring to a captive subsidiary and offshoring to a (large or medium-sized) arm’s-length outsourcing provider (Bunyaratavej et al., 2011; Doh, 2005; Lacity, Khan, & Yan, 2017; Mol & Brewster, 2014; Mudambi & Venzin, 2010).

Against this theoretical background, the emergence of microproviders from resource-poor countries presents a puzzle. How are they able to engage in market transactions with global clients? Such a market would seem poised to fail due to high barriers and low resources, and lead to either consolidation (i.e., larger firm size) or foreign acquisition (i.e., the MNE form). We posit that the answer to the puzzle lies in what the global online platforms do. Previous research has highlighted that platforms play an important role in reducing market frictions, especially the asymmetry of information between a prospective client and a provider concerning the quality of the provider (Galperin & Greppi, in press; Lin et al., 2016; Pallais, 2014; Tadelis, 2016). In the following sections, we model the three characteristics of the global platform economy—glocalization, platformization, individualization—from the perspective of the microprovider, asking what predicts the provider’s performance in this environment. We start with a simple TCE-consistent model of platforms as an alternative institution to employment in a local firm. However, TCE-style reasoning does not explain the precise mechanisms through which the platform institution manages to reduce the costs of international market access for microproviders. To address this, we adapt signaling theory (Connelly et al., 2011; Spence, 1974) to the model. Finally, we model the special implications of platform signaling for individualized emerging-economy providers facing liabilities related to their countries of origin.

### Glocalization as Reflected Through Reservation Wages

With the global platform economy a new offshoring institution, individuals have the option of offering their services to the global economy through platforms instead of through local (outsourcing) firms. In line with TCE, we posit that individuals will choose the most efficient institution; that is, they will choose the platform institution if and only if it better rewards them for their labor than the local labor market, whether in terms of monetary rewards, working conditions, or other compensation. We focus on pay for simplicity and model this choice using the notion of reservation wages (Lippman & McCall, 1976). This results in a model where differences in local labor market conditions are to some extent retained in the global platform economy, reflecting the glocalization thesis.

A reservation wage is defined as the lowest wage rate at which a worker would be willing to accept a particular type of job (for a survey of the literature, see Eckstein & Van den Berg, 2007). A central determinant of reservation wages is the best outside option for the worker: In conventional labor markets, the outside option is often interpreted as the wage that the worker is earning in the current job. For microproviders on global online platforms, we argue that the prevailing wage level on their local domestic labor market is a plausible approximation of their outside option, since their alternative is to seek employment locally.

Search theory predicts that reservation wages are particularly salient when there is intense competition (A. Manning, 2011). Platforms extend market access to providers around the world who would otherwise be excluded, likely intensifying competition. Although demand-side entry barriers are arguably also diminished (by, for instance, allowing outsourcing to be purchased in smaller chunks), which could mitigate competition, empirical evidence suggests that there is currently more supply than demand on global platforms (Graham et al., 2017; Kuek et al., 2015). When clients can select from many possible providers, providers have less room for negotiation, driving pay rates down toward the reservation wages. Eventually, providers in some countries will find it more lucrative to exercise their outside option. We thus hypothesize the following:

*Hypothesis 1:* There is a positive association between the pay rate of a microprovider on a global online platform and the local wage level in the home country.

### Platformization as a Signaling Environment

The platformization characteristic of the global platform economy entails that transactions are mediated through specialist websites instead of through peer-to-peer relationships taking place freely over the Internet. We suggested that this enables a reduction in transaction costs and, in particular, information asymmetry. While information asymmetry is to varying extents a concern in any real-world market (Akerlof, 1970), it is a particular concern in the IB context (Reuer & Ragozzino, 2014).

Foreign firms face information disadvantages compared with their domestic counterparts for several reasons: Foreign firms have inferior information about local market opportunities, organizations, and cultures, all of which contribute to the long-theorized liability of foreignness (e.g., Hymer, 1976; Zaheer, 1995). Information asymmetry tends to increase with geographic distance (Malhotra & Gaur, 2014). Conventional ways of signaling competence generally have lower value in a foreign country because it is hard to evaluate them (Barrett, McGuinness, & O’Brien, 2012; Oreopoulos, 2011). For instance, clients are likely to be largely unfamiliar with the quality and relative standing of educational institutions and firms listed in a foreign resumé.

The actions that firms take to mitigate the effects of information asymmetry in IB contexts can be modeled through signaling theory. Signaling theory was originally introduced by Spence (1974) to model the function of qualifications in a job market, but it is increasingly used as a general theory of behavior when two parties have access to different information (Bergh, Connelly, Ketchen, & Shannon, 2014; Connelly et al., 2011). One party (the signaler) has a characteristic that is important to the other party (the receiver), such as quality. The receiver cannot directly observe the quality, but the signaler can send signals to the receiver. If the signaling cost is correlated with the signaler’s quality (e.g., it is harder for low-quality firms to obtain quality management certifications; Terlaak & King, 2006), then it may be uneconomical for all but the highest-quality candidates to send signals, meaning that the signal has good *reliability* and thus reduces the receiver’s uncertainty about the signaler’s quality (Connelly et al., 2011).

In recent years, IB researchers have explicitly invoked signaling theory to show how foreign firms can assure investors of their quality through costly corporate governance arrangements (Bell et al., 2012), attest quality to potential international alliance partners through difficult-to-achieve affiliations with prominent financial institutions (Reuer & Ragozzino, 2014), choose the optimal entry mode (Stevens, Makarius, & Mukherjee, 2015), and signal information through business group characteristics (Mukherjee, Makarius, & Stevens, 2018). Though earlier studies on the liability of foreignness did not explicitly invoke signaling theory, they likewise identified the lack of company image or information on a foreign company in a new host country as one source of its liability of foreignness (Kostova & Zaheer, 1999; Zaheer, 1995) and proposed costly investments in social responsibility, organizational credibility, and local top management teams to mitigate it (Kostova & Zaheer, 1999; Luo et al., 2002; Mezias, 2002).

What the signaling strategies identified in earlier IB literature have in common is that they are appropriate for sizeable firms with considerable resources. Corporate governance arrangements, venture capital connections, and CSR activities are unlikely to be economical for even the highest-quality microenterprises. Even with good Internet connectivity, small firms comprising East Africa’s emerging business-process outsourcing (BPO) sector were found to lack the resources to build credibility directly with international clients (Foster et al., 2018). Microenterprises can set up their own websites to attempt to communicate their qualities to international clients, but such signals may be too cheap to carry much information and, moreover, suffer from lack of *signal observability*, or the extent to which receivers are able to notice such signals among millions of other websites (Connelly et al., 2001: 45). Cross-border business is typically dominated by larger firms with sufficient signaling resources, which may or may not use small firms as local subcontractors (S. Manning et al., 2017; United Nations Conference on Trade and Development, 2013).

We argue that the global platform economy is now changing this status quo, because it introduces new cross-border signaling mechanisms that are observable and economical for high-quality microproviders to use. The most basic way for microproviders to send signals through a platform is by posting self-generated evidence of quality. For instance, LinkedIn provides a field termed “education” where users can self-report their educational qualifications in a semistandardized form. Similarly, on online freelancing platforms, providers can typically list skills on their profile or even take computer-administered skill tests whose results are displayed on the profile. These signals have good observability, because the platform brings together potential clients and presents them with profiles with keywords that match search queries. However, the reliability of these signals is limited, because self-reported information can be inaccurate. Even computer-administered skill tests can be subverted by having another person take the test, referring to online “crib notes” that are available for many tests, or simply hiding a bad test result from one’s profile, when permitted by the platform. Nevertheless, it should still be easier for high-quality providers to put together impressive profiles and pass skill tests than for low-quality providers, so even these *unverified signals* should carry some information to prospective clients. We therefore hypothesize the following:

*Hypothesis 2a:* Unverified signals linked to a microprovider on a global online platform are positively associated with the pay rate.

Many platforms also enable the sending of signals that are in some way *verified* by the platform. Perhaps the best example of platform-verified information is reputation or feedback ratings, used prominently by Uber and online accommodation platforms. Individual service providers cannot provide reputation information about themselves; ratings represent the judgement of previous clients. Obtaining a good rating is presumably easier for high-quality providers than for low-quality providers, making the signal reliable. Ratings are still subject to various biases and dysfunctions, such as reciprocal behavior (Diekmann, Jann, Przepiorka, & Wehrli, 2014), inflation over time (Filippas, Horton, & Golden, 2018), and potentially even discrimination and blackmail (Resnick, Kuwabara, Zeckhauser, & Friedman, 2000). Ratings thus cannot be considered perfectly reliable quality signals, but platforms do make attempts to verify that they represent genuine feedback from previous clients. Indeed, previous research suggests that platform-verified reputation scores have a significant effect on outcomes (Galperin & Greppi, in press; Lin et al., 2016; Pallais, 2014). We therefore hypothesize the following:

*Hypothesis 2b:* Platform-verified signals linked to a microprovider on a global online platform are positively associated with pay rate, more so than unverified signals.

It is important to note that ratings still represent a judgement. A potential client cannot be sure of the criteria used by previous clients or whether they were particularly harsh or lenient. A third form of signaling in the global platform economy that largely eliminates such subjective biases is platform-generated signaling. For instance, a platform can observe and record the number of projects that a microprovider has completed. To the extent that experience enhances competence (Arrow, 1962), this is arguably an imperfect but nevertheless valid measure of provider quality. Moreover, it is highly reliable, since it is based on observation rather than reports by the providers themselves or by clients. Similar notions of observational, transactional, automatically generated data have been termed in other contexts as “digital trace data” or “big data” (Lazer & Radford, 2017). Regardless of label, we hypothesize that such platform-generated signals carry the most quality information and, consequently, the following:

*Hypothesis 2c:* Platform-generated signals linked to a microprovider on a global online platform are positively associated with pay rate, more so than platform-verified signals.

In summary, we posit that online platforms provide a new signaling environment where signals linked to microproviders are observable to international clients and that they enable a hierarchy of signaling mechanisms consisting of unverified signals, platform-verified signals, and platform-provided signals as the most reliable type. Understanding this platformization, or role of platforms as information intermediaries, is crucial to explaining microprovider performance in the global platform economy.

### Individualization Exacerbates Statistical Discrimination

It is increasingly recognized that some liabilities are associated not just with foreignness in general but with firms originating specifically from lower-income or emerging-economy countries (Pant & Ramachandran, 2012; Ramachandran & Pant, 2010). According to Ramachandran and Pant (2010: 240), MNEs’ national origins “mark them out not only in terms of their administrative heritage and idiosyncratic asset bundles, but also in terms of how they identify themselves and how their host country stakeholders regard them.” They argue that among the liabilities of origin that emerging-economy MNEs experience are negative perceptions attached to firms and products from those countries, long recognized in the international marketing literature (Johansson, Ronkainen, & Czinkota, 1994; Verlegh & Steenkamp, 1999). Building a strong brand for the firm or product has been found to mitigate the effects of negative country images and product-country images (Cordell, 1992; Johansson et al., 1994; Maheswaran, 1994).

We argue that this disadvantage applies to emerging-economy microproviders, as well, and may even be exacerbated due to the individualized nature of the global platform economy, where the country of origin of microproviders is typically prominently displayed to prospective clients. Since microproviders lack the resources of a larger company, they are unlikely to have established any sort of brand image in the prospective client’s country. The prospective client moreover has to choose from among numerous providers on the basis of a fairly limited set of signals, increasing the relative prominence of the country of origin.

We argue that the resulting effects on emerging-economy microprovider performance can be modeled through the notion of statistical discrimination. In contrast to the Beckerian notion of discrimination that suggests some form of preference for or animus against a given grouping (Becker, 1957), *statistical discrimination*, sometimes also called “rational discrimination,” is a technical term from labor economics that is used to highlight how an assumed statistical “average” is used to make decisions in the absence of adequate individual-level information (Ewens, Tomlin, & Wang, 2014). Statistical discrimination theory (Arrow, 1998; Phelps, 1972) predicts that stereotyping will occur when there is imperfect information and that the less information there is, the greater the role of stereotypes. Substantial previous work has found evidence of such stereotyping disadvantaging minorities and women in local labor markets (Altonji & Pierret, 2001; Lang & Manove, 2011; Pinkston, 2006; Tomaskovic-Devey & Skaggs, 1999). For the same mechanism to also affect microproviders from emerging-economy countries in global platforms is consistent with prior evidence. Indeed, Mill’s (2011) study of an online labor platform found that clients put more weight on a provider’s country of origin if the provider has no reputation information available.

Statistical discrimination theory moreover predicts that increases in individual-level information should reduce stereotyping. Two studies (Agrawal, Lacetera, & Lyons, 2013; Galperin & Greppi, in press) accordingly find that increases in platform-verified signals disproportionately benefit providers from lower-income countries. The mechanism according to statistical discrimination theory is that the “average” provider from a given lower-income country is seen as a riskier choice by clients, because there is more uncertainty about the quality, as the provider is drawn from a labor pool perceived as weaker or more mixed. Additional verified information about a provider’s quality consequently results in a substantial improvement in what they can earn. In contrast, hiring an “average” provider from a high-income country is assumed to be less risky by default, so additional information about them results in comparatively smaller adjustments. This leads to our final hypothesis:

*Hypothesis 3:* The positive association between platform-mediated quality signals and pay rate is stronger for emerging-economy microproviders.

In sum, we posit that microprovider pay rates in the global platform economy are partly a reflection of local wage levels and that emerging-economy microproviders face statistical discrimination as a result of clients’ difficulties in assessing large numbers of providers from many different countries. Both of these effects are also found in conventional labor and outsourcing markets, and result in depressed earnings for providers from lower-income countries. However, a key characteristic of the global platform economy is platformization, whereby online platforms mediate three different types of signals of provider quality: unverified, platform-verified, and platform-generated signals. All microproviders benefit from these quality signals, but emerging-economy microproviders benefit from them more, because they displace stereotypes that clients apply on the basis of the emerging-economy country of origin. The next section describes a research design to test these hypotheses empirically.

## Research Design

Our empirical approach is based on a mixed-methods design involving digital trace data and qualitative interview data. Both data sets come from a multiyear research program on online labor platforms in emerging-economy countries. The main method is regression analysis of variables derived from the digital trace data. A strong justification for the use of digital trace data in social science research is that phenomena under study are themselves becoming increasingly digitalized: “Social life increasingly occurs in digital environments and continues to be mediated by digital systems. Big data represent the data being generated by the digitization of social life” (Lazer & Radford, 2017: 19). This is especially true of the global platform economy, one of the constituent characteristics of which we argued is platformization, or the intermediation of transactions by digital platforms. The interview data provide a different set of methodological affordances that are used to “offset” some of the limitations of the trace data (Bryman, 2006). Findings are ultimately synthesized into a “negotiated account” that involves both convergent validation (triangulation) and augmenting one method’s deficiencies with the other (Fielding, 2012).

### Main Data: Digital Trace Data From a Leading Platform

Our digital trace data come from a leading global online labor platform, which did not wish to be identified by name. The platform matches microproviders with clients and facilitates the entire contracting relationship from search and negotiation to supervision, delivery, billing, and postproject evaluation. Supervision on the platform is facilitated by means such as periodic screen captures of the provider’s computer. Payment is enforced via means such as escrow. This makes it less risky for firms and individuals to enter into contracts with counterparties with whom they have had no previous contact. The platform approximates a double auction model, where both clients and providers are able to make offers and therefore influence pay rates; the platform charges a fee of approximately 10%. The platform provides an excellent context for this study because it hosts clients and providers from any country and is among the largest of such marketplaces (Kuek et al., 2015).

Our data are derived from the transaction records of all projects mediated by the platform from March 1 to August 31, 2013, provided to us by the platform company in an anonymized form. The use of such trace data or “big data” is still relatively new in the social sciences and has provoked much enthusiasm. Some of its frequently cited strengths are that it is microlevel yet offers macrocoverage, capable of smoothly transcending geographic boundaries, continuous in time rather than consisting of a series of snapshots, and observational rather than self-reported (Hampton, 2017; Lazer et al., 2009). By offering unobtrusive observational measures of large-scale social phenomena, digital trace data can avoid a host of systemic biases common in social science data (Lazer & Radford, 2017). According to Golder and Macy (2014: 131), “these methods greatly expand our ability to measure changes in behavior, not just opinion; to measure these changes at the individual level yet on a global scale.”

However, it is increasingly clear that the use of digital trace data also comes with serious pitfalls and limitations. One pitfall is that the variables in trace data are not designed by researchers but generated as “digital exhausts” from commercial processes (Lazer & Radford, 2017; Schober, Pasek, Guggenheim, Lampe, & Conrad, 2016). They are not previously validated measures of theoretical concepts and thus risk being convenience variables of limited validity. A related pitfall is apophenia, or “seeing patterns where none actually exist” (Boyd & Crawford, 2012: 668). Spurious correlations become more likely with large numbers of variables, and artefacts can appear from the hidden technical processes that generate the data. Both pitfalls are exacerbated in typical computational social science approaches to trace data analysis, because they are data driven and often reject hypothesis testing, making few assumptions about which variables are relevant and instead aiming to let the data “speak for itself” (Hampton, 2017; Schober et al., 2016).

We address these pitfalls by adopting a theory-driven approach to using trace data. Variables are selected on the basis of their links to concepts in the theoretical model, and we reach outside the trace data for an additional variable when necessary. Specifically, to address Hypothesis 1, the local wage level is measured as the country’s average hourly wage across all sectors, calculated from the Occupation Wages Around the World (OWW) database (Oostendorp, 2012). The variable is expressed in U.S. dollars, converted using exchange rates (rather than purchasing power parity [PPP]) to correspond with the method a provider would use to compare local wages and online rates. The most recent year for which wage data could be obtained across the countries is 2008. Competition is operationalized as the number of applicants, that is, the number of competing applications for each of the projects completed by a provider, derived from the trace data.

Finding valid measures of signaling for testing Hypothesis 2 is greatly assisted by the fact that some of the variables in the trace data are co-constitutive of the signaling processes that we are modeling; that is, they are the same variables that clients observe on the platform. Platform-generated signaling is thus operationalized as experience, or the number of projects the provider has completed on the platform since joining it. Platform-verified signaling is operationalized as reputation, the provider’s mean feedback score from clients on a scale of 0 to 5. Unverified signaling is operationalized as skill tests, the number of skill tests administered by the platform that the provider has completed and published on their profile. The tests are voluntary and measure such skills as typing, language proficiency, and office software use. Unverified signaling also includes English skills, the providers’ self-reported skill in English measured from 0 to 5. Hypothesis 3 is addressed with the same independent variables. Table 1 shows the variables used and their descriptive statistics. To enhance interpretability, at the analysis stage, all variables are standardized by subtracting means and dividing by standard deviations.

**Table 1:** Means (Standard Deviations) of Main Variables of Interest

Variable	Writing		Graphic Design	
Hourly rate (USD)	11.04	(10.73)	11.06	(9.15)
Local mean wage (USD)	9.02	(9.04)	3.73	(6.70)
Experience	62.23	(81.49)	90.33	(110.84)
Reputation	4.59	(0.85)	4.66	(0.71)
Skill tests	4.41	(5.83)	4.10	(5.00)
English skill	4.96	(0.28)	4.83	(0.48)
Number of applicants	19.76	(19.55)	21.60	(26.22)
Sample size	4,927		5,140	

The dependent variable, pay rate, is operationalized as the hourly pay to the project’s provider in U.S. dollars. The platform also supports fixed-payment projects, but controlling project size becomes a problem in these. To eliminate this major source of variation, only hourly-paying projects (49%) were selected for analysis. As is common in studies of wages and earnings, hourly pay is right-skewed and is entered into regression specifications log-transformed.

To further ensure that we are comparing “apples with apples” when it comes to country differences, out of all the diverse types of projects contracted via the platform, we chose to focus on two types: writing and graphic-design projects. Writing projects are defined as projects categorized in “Blog and Article Writing,” “Creative Writing,” “Copywriting,” and “Technical Writing” in the platform’s ontology (34,352 projects in total). Graphic-design projects are projects classified in the “Graphic Design” category (25,814 projects). Programming work, for instance, is much more heterogeneous, greatly increasing potential unobserved sources of variation. Moreover, we chose writing because it was what many of our interview participants were doing, requires no formal qualifications, is supplied by providers from around the world, and exists in sufficient numbers in the trace data to provide good statistical power. We chose graphic design because it has these same useful characteristics (except that it was not done by interview participants) and yet requires a different set of skills. In particular, it is much less dependent on language skills.

To eliminate variation in host country effects across different client countries, only projects where the client is from the United States or Canada were included. We chose U.S. and Canadian buyers because their combined market share is very high in the online gig economy (Kässi & Lehdonvirta, 2016). Projects with an undefined or zero hourly rate and projects where no money was charged were cut. Finally, since OWW wage data were not available for all provider countries, some cases had to be dropped. After all these criteria were applied, 4,927 writing projects and 5,140 graphic-design projects were brought forward for analysis.

### Complementary Data: Interviews With Emerging-Economy Microproviders

A further pitfall of digital trace data research is the issue of generalizability from one platform to other platforms and populations (Hampton, 2017; Lazer & Radford, 2017). Trace data can produce a “case study” of a single platform with excellent internal validity but afford few ways of assessing the results’ external validity. Earlier research suggests that microproviders use multiple global platforms to access international demand (Graham et al., 2017) and that platform design is somewhat isomorphic, especially when it comes to signaling mechanisms (Tadelis, 2016). There are thus reasons to expect that our model and findings from one platform and two types of work would apply to the global platform economy more generally. But to provide empirical support for the generalizability of trace data findings, Lazer and Radford (2017) suggest using data from multiple sources. We introduce supplementary qualitative data from interviews with emerging-economy microproviders, covering a range of different platforms and types of work.

The semistructured face-to-face interviews were conducted by the authors in six countries between September 2014 and December 2015. In total, 107 microproviders were interviewed across Southeast Asia (in the Philippines, Vietnam, and Malaysia) and Sub-Saharan Africa (in South Africa, Kenya, and Nigeria). Participants were recruited through four online labor platforms, including the platform providing the trace data. The majority of interview participants engaged with tasks such as data entry, blog and article writing, virtual assistant services, search engine optimization, and audio transcription. Most interviews lasted around 1.5 hr and were recorded, transcribed, and coded in NVivo. The sampling criteria, recruitment strategy, interview protocol, and qualitative analysis approach of the study are detailed in the appendix. The aim of our qualitative analysis is not to try to replicate the findings of the quantitative analysis but to construct a parallel account of the phenomenon from a different epistemological starting point.

The qualitative analysis also allows us to address another set of critiques of trace data research: the need for social context in interpreting data (Neff, Tanweer, Fiore-Gartland, & Osburn, 2017). To make accurate inferences, researchers need qualitative understandings of the sociotechnical arrangements that produce the data. Data are never value free and can reflect assumptions, interpretations, and negotiation on the part of platform designers and other stakeholders. The interview data allow us to see how microproviders themselves experience the dynamics that the trace data depict and to check our assumptions accordingly. To achieve this, it is important that the qualitative analysis remain relatively open-ended instead of attempting to replicate the deductive logic underpinning hypotheses testing.

The findings are presented so that each of the quantitative analyses is followed by qualitative findings on the same dynamic. The two data sources provide distinct, nonoverlapping affordances for drawing conclusions: The trace data provide unbiased observational measures and formal generalizability to a limited population, while the qualitative data provide rich, noisy measures covering a wide range of cases but lacking formal generalizability. The objective of the mixed-methods approach is therefore not simply to strengthen the reliability of our conclusions through convergent validation (triangulation) but to increase the “analytic density” of the research (Fielding, 2012), helping to extend the scope and depth of the conclusions by “offsetting” one data source’s weaknesses with the other (Bryman, 2006). Integrating the findings is thus a matter of constructing a “negotiated account” that compares and contrasts the findings from the different methods. This is necessarily methodologically innovative, as there is no best practice for integrating mixed-methods findings (Fielding, 2012), especially for the relatively novel combination of digital trace data and semistructured interviews (Eynon, Hjorth, Yasseri, & Gillani, 2016).

## Results

### Glocalization Through Reservation Wages

#### Quantitative findings

To formally test Hypotheses 1 and 2a through 2c, we estimate variants of the following model:

(1) $$y_{ik}=\alpha +Z_{i}^{"}\Gamma +\beta localwage_{k}+\delta NumApplicants_{ik}+\epsilon_{ik}$$

where $y_{ik}$ is the project-specific hourly wage for worker *i* from country *k*, and $Z_{i}^{"}\Gamma$ comprises the platform-mediated signals on provider competence (experience, reputation, skill tests, and English skills, all standardized). The results are summarized in Table 2.

**Table 2:** Predictors of Hourly Pay (Dependent Variable: Hourly Rate [Log])

Predictor	(1) Writing	(2) Design	(3) Writing	(4) Design	(5) Writing	(6) Design
Local mean wage (log)	0.32 (0.01)	0.26 (0.01)	0.32 (0.01)	0.27 (0.01)	0.33 (0.01)	0.27 (0.01)
	*t* = 48.86	*t* = 35.73	*t* = 50.35	*t* = 36.67	*t* = 44.91	*t* = 35.51
	*p* = .000	*p* = .000	*p* = .000	*p* = .000	*p* = .000	*p* = .000
Number of applicants (log)	−0.09 (0.01)	−0.09 (0.01)	−0.08 (0.01)	−0.08 (0.01)	−0.08 (0.01)	−0.08 (0.01)
	*t* = −10.99	*t* = −13.30	*t* = −9.56	*t* = −12.50	*t* = −9.74	*t* = −12.51
	*p* = .000	*p* = .000	*p* = .000	*p* = .000	*p* = .000	*p* = .000
Experience			0.13 (0.01)	0.06 (0.01)	0.17 (0.02)	0.06 (0.01)
			*t* = 10.56	*t* = 7.66	*t* = 11.39	*t* = 7.45
			*p* = .000	*p* = .000	*p* = .000	*p* = .000
Reputation			0.09 (0.011)	0.06 (0.01)	0.12 (0.015)	0.06 (0.01)
			*t* = 7.99	*t* = 4.67	*t* = 7.98	*t* = 4.33
			*p* = .000	*p* = .000	*p* = .000	*p* = .000
Skill tests			0.07 (0.01)	0.02 (0.01)	0.10 (0.01)	0.03 (0.01)
			*t* = 6.31	*t* = 1.98	*t* = 7.14	*t* = 2.29
			*p* = .000	*p* = .048	*p* = .000	*p* = .022
English skills			0.05 (0.02)	0.04 (0.01)	0.09 (0.02)	0.04 (0.01)
			*t* = 2.66	*t* = 3.31	*t* = 3.80	*t* = 3.34
			*p* = .008	*p* = .001	*p* = .000	*p* = .001
Experience × Local Mean Wage					−0.03 (0.01)	−0.02 (0.01)
					*t* = −4.48	*t* = −2.76
					*p* = .000	*p* = .006
Reputation × Local Mean Wage					−0.019 (0.007)	−0.000 (0.008)
					*t* = −2.95	*t* = −0.02
					*p* = .004	*p* = .984
Skill Tests × Local Mean Wage					−0.02 (0.01)	0.01 (0.01)
					*t* = −2.68	*t* = 1.32
					*p* = .008	*p* = .188
English Skills × Local Mean Wage					−0.04 (0.01)	−0.00 (0.01)
					*t* = −2.94	*t* = −0.14
					*p* = .004	*p* = .892
Constant	1.83 (0.024)	2.20 (0.02)	1.78 (0.02)	2.16 (0.02)	1.78 (0.02)	2.16 (0.02)
	*t* = 75.75	*t* = 125.74	*t* = 75.03	*t* = 121.34	*t* = 74.61	*t* = 120.74
	*p* = .000	*p* = .000	*p* = .000	*p* = .000	*p* = .000	*p* = .00
Observations	4,927	5,140	4,927	5,140	4,927	5,140
*R* ^2^	.35	.24	.40	.26	.40	.26

*Note*: Regression coefficients, standard deviations in parentheses, *t* statistics, and *p* values.

The hypothesis that there is a relationship between the pay rate of a microprovider on the online platform and the local wage level in the home country (Hypothesis 1) is tested in its simplest form in columns 1 and 2 of Table 2. The results show that the level of competition has a highly significant effect, and as hypothesized, the association between local wage level and earnings is positive and statistically significant. The point estimates of 0.322 and 0.263 imply that a one-dollar increase in the local wage level is associated with a 32-cent increase in the hourly pay rate earned from writing projects and a 26-cent increase in graphic-design projects.

**Figure 1:**
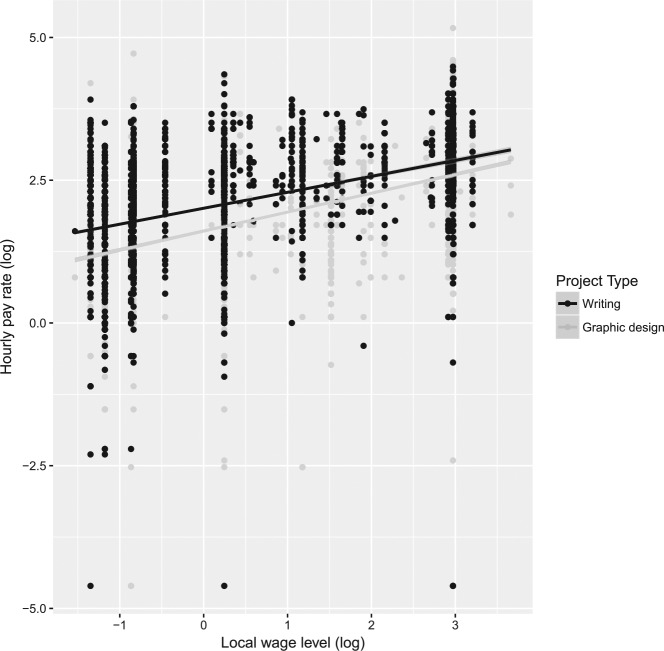
Association Between Local Wage Levels and Online Hourly Rates

However, the results do not imply that local wage levels fully account for providers’ online earnings—far from it. Competition and local wage levels together account for only approximately 35% of the variation in writing pay rates and 24% in graphic-design pay rates. This is illustrated in Figure 1, which plots local wage levels against hourly rates. The differences in pay rates between individual observations in countries with the same local wage levels in the plot remain substantial.

#### Qualitative findings

Our interview participants all used global platforms to access foreign demand, but most had also previously worked or were concurrently working in the local labor market. For instance, Ezekiel (male, 26, Nairobi, Kenya; all names are pseudonyms) was working at a local BPO office specializing in audio transcription when a friend introduced him to a global platform. He started moonlighting through the platform as a freelance transcriber from his BPO office after regular working hours and from his home. After a while, he quit the BPO job and started working exclusively through platforms, because he felt it paid more and gave him more flexibility. Others shared similar stories:

The convenience of staying at home and doing exactly the same thing and being paid much better than getting an office job, it kind of like really appealed to me. (female, late 20s, Manila, Philippines)

In contrast, Rica and Joshua (early 30s, Manila, Philippines) are a couple who both worked through a global platform from their home but also concurrently held full-time jobs at a local BPO office. They offered similar services through the platform as they performed in their BPO jobs, centering on finding contact details from the web and entering information into databases to support international sales teams. They had a baby daughter and held on to their regular jobs for income security; platform clients paid well, but projects sometimes ended abruptly:

There’s no security of tenure on [a platform]. If your client wakes up on the wrong side of the bed today, and you did not meet the deadline, you get flat out—they’ll be gone. (Joshua, early 30s, Manila, Philippines)

In line with our model, interview participants thus conceived of the global platform economy as an alternative institution to their local labor markets and often expressed clear rationales for choosing between microprovidership and local employment. Even interviewees who had become microproviders straight from college and had thus never experienced local employment could articulate rationales for the choice. The rationales included differences in pay but also in income security, future earnings potential, flexibility, variety, and other job quality characteristics. Interview participants in large cities with poor transport infrastructure highlighted the benefits of avoiding commutes. The downsides of microprovidership that drove participants to local employment included unpredictable earnings but also lack of social contact and asocial working hours, as many worked during international clients’ daytime hours. The novelty of the platform institution was also reflected in the fact that many participants experienced challenges in explaining their microprovider activities to family and friends:

When some [of] my neighbours, they ask me, “What do you do?” I tell them, I work online. They don’t understand. They ask me, “What do you buy?” I don’t buy. Yeah. They don’t understand what I [do for] work. (male, early 30s, Ho Chi Minh City, Vietnam)

The interviews show that the choice between platforms and local employment was also shaped by individuals’ positions in social structures. For instance, Helen (female, early 30s, Johannesburg, South Africa) switched to platform-based writing work from a regular job as a newspaper editor, because the latter was not compatible with what was expected of her as a mother. Other microproviders similarly turned to online work because of parenthood:

The big thing for me was I didn’t want to leave her [a young child] somewhere. I’m not one of those moms that would just go drop her off at a nursery. I do the attachment parenting thing. (female, mid-20s, Johannesburg, South Africa)

Jonalyn (female, late 20s, Manila, Philippines) alternated between temporary BPO jobs and microprovidership because of the difficulty of securing long-term BPO employment without college education. Three participants lacked work permits in the countries where they lived, excluding them from local formal employment entirely. Local social structures as well as individual agency thus played a part in determining which institutions individuals ended up working through, generating local variations in the supply of labor to the global platform economy that reflected differences in labor market conditions but also other social and cultural idiosyncrasies.

### Platformization as a Signaling Environment

#### Quantitative findings

Next, we turn to the proposed mechanisms through which platforms reduce information asymmetries and make cross-border exchange possible. Columns 3 and 4 of Table 2 report the effects of platform-mediated quality signals on provider pay rates. We hypothesized that platform-mediated signals will improve earnings and also that the more reliable the signals, the greater the effect (Hypotheses 2a through 2c).

We find that unverified signals are associated with the smallest effects. Skill tests have a coefficient of 0.074 for writing and 0.021 for graphic design, and self-reported English skills 0.049 for writing and 0.037 for graphic design. In practical terms, this means that a one-standard-deviation increase in skill tests is associated with an approximately 7% rate increase in writing tasks and a 2% rate increase in graphic design tasks. These are followed by reputation, a platform-verified signal, with an effect of 0.087 for writing tasks and 0.057 for graphic design. One-sided *t* tests comparing the parameter estimates of skill tests against reputation produce *p* values of 0.20 (*t* = 0.82, *df* = 4926) for writing and 0.018 (*t* = 2.47, *df* = 5139) for graphic design.

In terms of both coefficients and significance levels, the greatest effect is found for experience, which represents platform-generated signaling, the most reliable type. Its effect is 0.129 in writing work and 0.062 in graphic design, corresponding to rate increases of approximately 13% and 6% per standard deviation. One-sided *t* tests comparing these estimates against the effects of reputation produce *p* values of 0.00 (*t* = 2.43, *df* = 4926) for writing and 0.33 (*t* = 0.43, *df* = 5139) for graphic design. One-sided *t* tests comparing the estimates against skill tests produce *p* values of 0.00 for both types of work (*t* = 3.17, *df* = 4926; *t* = 3.59, *df* = 5139).

The findings show that the effect sizes for reputation and skill tests are larger in both practical and statistical terms in writing work than they are in graphic design. This divergence is not attributable to differences in statistical power, as sample sizes and estimated standard errors are similar across the two specifications. It may be that clients rely somewhat less on platform-mediated signals when hiring designers than when hiring writers. This could be explained by the fact that graphic designers typically provide a portfolio of their work as an additional signal, while platform-based writers typically do not. Nevertheless, the directions and magnitudes of the parameter estimates all align with our theoretical predictions and are of a practically significant size. Four of the six comparisons, examining which signals are the most influential, are statistically significant on conventional significance levels. We interpret these findings as broadly supportive of Hypotheses 2a through 2c.

#### Qualitative findings

All of our interviews point to platform-mediated signals as being crucial for microprovider success. Newer microproviders were eagerly trying to strengthen the quality signals their profiles were sending; more established providers recognized the value of their profiles’ quality signals and likewise made efforts to maintain and improve them.

So when it comes to setting rates . . . I think it’s all trickles down to how your profile looks like, because it shows the sense of seriousness about yourself. (male, 25, Nairobi, Kenya)

Once you’re able to take a test for that skill you’ve acquired, you’re telling your potential clients that I’m capable of doing this stuff: “Hey you can go see my profile; I’m capable of doing that.” (male, 23, Abuja, Nigeria)

Participants frequently spoke about the influence that signals had on both pay rates and the likelihood of winning contracts. Experienced microproviders whose profiles sent strong quality signals felt empowered to increase their rates and experienced less impact from competition:

Since I had good ratings, I got invitations—many invitations, and many inquiries. I could also get my rates a bit higher. (female, early 30s, Manila, Philippines)

I ask for more money now than I asked for 5 years ago. I have more experience, and . . . many good feedbacks and clients can trust me on projects. (male, 31, Ho Chi Minh City, Vietnam)

I [effectively] choose how much I get paid. I have got to that level where I have sufficient experience and sufficient feedback to set my own rates. . . . I am grouped [by the platform] under expert. (male, 30, Nairobi, Kenya)

Participants to some extent distinguished between different types of signals, and a few explicitly judged what we termed unverified signals as less effective than platform-verified and platform-generated signals.

You may say I attended Harvard, I have this, I have that and other. Nobody is going to pay you based on that. . . . While your skills are very important and all that, the validation comes from the client feedback. The higher the client feedback, the higher the chances you [will succeed]. (male, 36, Lagos, Nigeria)

Overall, interview participants tended to put most emphasis on client feedback (reputation) ratings. They were seen as a powerful but somewhat noisy signal: Clients might forget to leave feedback, leave unfair feedback, or even use feedback as a means to extort the provider. Since ratings were seen as such a powerful signal, participants attempted to manage them in various ways, such as negotiating reciprocal five-star ratings with a client, forgoing payment to avoid a bad rating, complaining to platform customer support about unduly low ratings, starting afresh with a new account if bad feedback piled up, and assessing a client’s past feedback-giving behavior before accepting a contract:

I put so much effort into finding the right clients. . . . I look at their reviews, and if they don’t give good reviews back, I won’t even go for them, you know? I think you have to do the due diligence beforehand. (female, 59, Johannesburg, South Africa)

The feedback system thus structured practices in the market, but not always in ways that would leave an accurate trace in the feedback data; some practices were adopted to *avoid* leaving feedback data. Some participants also analyzed other successful providers’ data to derive insights on how they should improve their own signaling or observed competitors’ signals in bidding-contest situations to inform their own strategy. In this way, platform-mediated signals facilitated the learning and socialization of new microproviders into the global market.

You have to know what’s happening in the market. You visit other peoples’ profiles, you see you worked for this guy and he did this project. How much did he earn per article? You just . . . you have to at least watch other people what they’re doing, otherwise you’ll be left behind. (male, 27, Nairobi, Kenya)

There are people out there that have enormous [platform] profiles. . . . They’ve got thousands of hours under their belt and they’re writers, they’re editors and proofreaders and they’ve really done well. . . . I go, “Wow!” (female, 50, Cape Town, South Africa)

The qualitative evidence thus validates the idea of the platform as a signaling environment that reduces information asymmetry between clients and providers, and also suggests that signaling mechanisms are adopted as part of broader sociotechnical practices that enable and structure the global platform economy, with the consequence that their influence and importance extend beyond what is visible in the trace data.

### Individualization and Statistical Discrimination

#### Quantitative findings

The individualized nature of the global platform economy opens up the possibility of statistical discrimination, which would entail clients basing their inferences of provider quality on limited evidence and stereotypes, and providers having to convince clients of their skills. Our final hypothesis is that signals mediated by the platform can offset country-based stereotypes attached to providers, so that the pay rates of statistically discriminated-against providers catch up as platform-mediated evidence of competence increases (Hypothesis 3). We examine this by estimating the following linear regression model:

(2) $$\begin{array}{l} y_{ik}=\alpha +\beta_{1}localwage_{k}+\beta_{2}experience_{i}+\beta_{3}reputation_{i}+\beta_{4}skillcount_{i}\\ +\beta_{4}english_{i}+\delta NumApplicants_{ik}+\gamma_{1}\left(localwage_{k}\times experience_{i}\right)\\ +\gamma_{2}\left(localwage_{k}\times reputation_{i}\right)+\gamma_{3}\left(localwage_{k}\times \;skillcount_{i}\right)\\ +\gamma_{4}\left(localwage_{k}\times \;english_{i}\right)+\epsilon_{ik} \end{array}$$

We specifically examine whether the regression coefficients on interaction terms $\left(localwage_{k}\;\times \;experience_{i}\right),\;\left(localwage_{k}\;\times \;reputation_{i}\right),\;\left(localwage_{k}\times skillcount_{i}\right)$, and $\left(localwage_{k}\times english_{i}\right)$ are negative and statistically significant. A negative coefficient on the interaction term implies that providers from countries with lower local wages experience larger increases in pay (relative to counterparts from higher-income countries) when they gain work experience, get good feedback, complete skill tests, or report having good English skills.

Table 2 presents the estimation results for writing work (column 5) and graphic design (column 6). The interaction terms estimated from the writing data are consistently negative and statistically different from zero. Moreover, they support the notion that more reliable types of signaling have a greater effect. Both when considering the size of the coefficient and the significance levels, the greatest reduction of statistical discrimination is associated with platform-generated signals (experience), followed by platform-verified signals (reputation) and, finally, unverified signals (skill tests and self-reported English skills). For the graphics projects subset of the data, only the $\left(localwage_{k}\times experience_{i}\right)$ interaction term differs from zero in conventional risk levels. In other words, only platform-generated signaling helped to overcome statistical discrimination in graphic design. The size of this effect is similar in both types of work.

To illustrate the practical significance of the results, we can observe that Filipino providers doing writing work earn a mean of $6.2 per hour. Their marginal return to gaining one extra project’s worth of platform-generated experience signals is 16%. Writing providers from the United States, on the other hand, earn an average of $19.5 per hour, and their marginal return to one more project is about 7%. An average Filipino graphic-design provider earns $8.47 per hour and has a 5% marginal return for experience. The corresponding numbers for graphic designers from the United States are $19.53 and 0.1%. Platform-mediated signaling thus considerably reduces the pay gap between similar providers from low- and high-income countries but is unlikely to eradicate it completely. Specifications in columns 5 and 6 of Table 2 imply that a provider from the lowest-income country would need to signal around a thousand projects’ worth of experience on the platform to obtain the same rates as a provider from the highest-income country.

#### Qualitative findings

Offering convergent validity to our model, many interview participants expressed that they were being disadvantaged by clients’ lack of knowledge about their emerging-economy home countries’ institutions and education systems and by the negative stereotypes attached to their countries. They felt that this was making it more difficult for them to win contracts and charge higher rates. This aspect of the global platform economy was sometimes felt to be the most difficult one to deal with.

I think people misunderstand the region, misunderstand the people, misunderstand the education. Not everybody in South Africa is poor or will accept a substandard wage. . . . You could go so far as to call it borderline ignorance. . . . We have roads. We have infrastructure. We have universities, but a lot of people don’t know this. (female, 36, Johannesburg, South Africa)

[Clients] feel like Africa is just one country. They will decide based on that perception, not because of what they researched or what they know. It is easy for them to draw conclusions that I don’t think this guy really knows what he’s doing. (male, 31, Lagos, Nigeria)

This perception [of clients] that third-world countries are not good. It hurts. It’s very demoralizing. The perception, I really hate it because in this country we’ve come up with some of the most innovative ideas and if people in [a leading platform] would just utilize such talents, they’re here. (male, 27, Nairobi, Kenya)

These participants’ experiences are consistent with the statistical discrimination model, where the absence of accurate information leads to reliance on stereotypes. Participants from English-speaking countries argued that this stereotyping extended to language skills:

There’s also the perception that the quality will not be as good, the English language skills will not be as good, and for some people that holds true. A lot of people on this continent, that’s not true at all. We speak, read, and write excellent English. If you’ve been taught English since the age of 3, and you do British exams . . . all you need is just that opportunity. (female, 36, Johannesburg, South Africa)

There is a bias, I think . . . especially with transcription. I think like graphic design or those other things would not be as much but transcription you have to have good English. I don’t think [clients] think that we have good English. . . . There’s just that whole perception of Africa is not as developed. . . . People [clients] will not understand why I say I’m a native English speaker but I am from Africa. . . . English is my first language. . . . Oh my gosh, we can speak English in Africa. We speak English quite well! (female, 21, Nairobi, Kenya)

Some people assume Kenyans don’t speak English or something like that . . . so you have to really prove yourself on that end. (female, 24, Nairobi, Kenya)

Microproviders might of course sometimes mistakenly attribute their own failures to statistical discrimination. But as discussed in the previous section, participants generally saw that platform-mediated quality signals had a very significant positive impact on their pay rates and likelihoods of winning contracts. Consistent with Hypothesis 3, a few participants drew an explicit causal link between an increase in such platform-mediated signaling and a reduction in the negative stereotyping they experienced:

Once . . . you do get those first few projects and you get good feedback for that, then it doesn’t really matter anymore that you’re from the Philippines. (male, late 20s, Manila, Philippines)

As at then I had almost 250 jobs and all of them were five stars. . . . Most of the time, when I send the invitations, they say, “Hey, I see your profile and I see you’re a very good programmer, can you help me with this?” They don’t say, “Hey, I see your profile, you’re a Nigerian.” (male, 29, Lagos, Nigeria)

Actually when Jack, my [platform] employer saw this, this is what he said in the e-mail he sent me: “Kingsley, your profile is even better than the Americans.” (male, 25, Nairobi, Kenya)

Another strategy that participants used to mitigate negative stereotyping was to attempt to build longer-term relationships with clients. The more a provider worked for a single client, the more informed that client became about the provider’s skills and competence, reducing the client’s reliance on stereotyping. This could entail trading away some of the benefits of microprovidership, such as flexibility, and could take on characteristics of conventional employment.

## Discussion

In this section, we provide a “negotiated account,” a synthesis of the quantitative and qualitative findings, and situate them in the context of IB literature to draw out theoretical contributions.

According to TCE-based theories of global sourcing, the two established modes of service offshoring—outsourcing to service provider firms and offshoring to captive subsidiaries—arose as institutional responses to different transaction cost landscapes: Whenever gains from cross-border exchange could not be realized economically via arm’s-length markets, they were realized by internalizing the exchange with a subsidiary or similar entry mode (Buckley & Casson, 2009; Bunyaratavej et al., 2011; Chen, 2010). These two offshoring modes were both enabled by the adoption of information and communication technology that made service offshoring economical in the first place, launching the 1990s “tradability revolution” (Doh, 2005; Dossani & Kenney, 2007). Now, thanks to what may in time come to be regarded as a second tradability revolution, the rise of personal Internet access and global online platforms is once again changing the cost landscape and redefining what is possible. Our findings show that the global platform economy has emerged as a new institutional form for realizing gains from cross-border exchange. It gives individuals the option of directly addressing foreign demand for their skills and labor, becoming microproviders instead of seeking employment at a local outsourcing provider company or MNE subsidiary. Moreover, the opportunities can be accessed even in locations where conventional offshoring institutions are not present.

According to our model, consistent with TCE, microproviders choose this novel institution to the extent that it better rewards their labor than firms in the local labor market do, and we indeed found that local wage levels are a significant predictor of microproviders’ platform pay rates (Hypothesis 1). Qualitative findings likewise supported the notion that individuals made choices between platforms and local employment based on pay as well as broader outcomes, such as working hours, income security, and potential for income growth. Despite being a global phenomenon, the platform economy thus to some extent retains and reproduces highly localized differences in pay and other labor market conditions, allowing clients to benefit from them. This “glocalization” stands in slight contrast to conventional offshoring, which is likewise motivated by location advantages yet constrained by infrastructure, regulation, and other factors to cluster into relatively much smaller numbers of locations (Gereffi & Lee, 2016; S. Manning, 2013).

Some participants simultaneously undertook a mix of microprovider projects through a platform and regular employment at a local BPO company. This suggests that global platforms may in some situations complement rather than substitute for conventional offshoring institutions. At this stage, platforms are an efficient institution for offshoring small amounts of work, one-off projects, and projects with tight turnaround times (Corporaal & Lehdonvirta, 2017), whereas conventional offshoring institutions seem more suited for the large-scale and continuous offshoring of business functions (S. Manning et al., 2017). But already, global platforms seem to substitute conventional offshoring institutions for some types of work, and as with conventional offshoring (Lewin & Peeters, 2006), this transformation is likely to continue.

By adding signaling theory (Connelly et al., 2011; Spence, 1974) to the TCE model, we were able to provide important detail on platformization, or the mechanisms through which the platform institution makes it economical for microproviders to access foreign demand. We showed that the platform provides mechanisms for microproviders to signal their quality to prospective buyers, reducing the information asymmetry that would otherwise prevent the exchange. These platform-provided signaling mechanisms are much less costly than cross-border signaling mechanisms identified in earlier IB literature (e.g., Bell et al., 2012; Mukherjee et al., 2018; Reuer & Ragozzino, 2014) and therefore accessible to microproviders. Crucially, they are less costly for high-quality than for low-quality providers and are thus able to carry real quality information (as evidenced by how they influenced pay rates). We proposed three different types of platform-mediated signals that differ in how reliable they are, based on how difficult it is to send dishonest signals: unverified signals, platform-verified signals, and platform-generated signals. Consistent with signaling theory, we found that the greater the proposed reliability of the signal, the greater the effect it had on pay rates, with platform-generated signals having the greatest effect (Hypotheses 2a though 2c).

This finding has practical implications for microprovider strategy. Platform-generated signals (like experience) have a stronger effect than the more visible (and often more controversial) feedback ratings, which are platform-verified signals. Providers may be overinvesting in managing platform-verified signals and underinvesting in maximizing platform-generated signals by doing too few projects too well. Moreover, the significant differences in the three signaling mechanisms’ efficacy suggest that further developments in platform technology could yield even more effective mechanisms. Connelly and colleagues (2011) suggest that signal reliability has two subcomponents: *signal honesty* and *signal fit*. Signal honesty can be understood as the degree to which the signal correlates with the characteristic that it purports to represent, while signal fit can be understood as the degree to which the signal is correlated with the characteristic that matters for the receiver. For example, a platform-generated measure of projects completed is an honest signal, as it accurately represents the number of projects a provider has completed, but is only a partial fit with provider quality, the characteristic that matters for clients. Platforms could thus further reduce information asymmetry by improving honesty and fit. More precise observational measures of experience (perhaps such as measures of experience with specific software tools or technologies that are important to clients) could improve the fit of the platform-generated signaling, while more stringent skill tests could improve the honesty of the skill test signal and allow us to reclassify it from unverified to platform verified.

Our findings also explain how the platform institution helps emerging-economy microproviders access global demand, thus enriching the literature on internationalization from emerging economies. Participants from those economies are doubly disadvantaged in global markets, not only by the information asymmetries inherent in any cross-border business but also by negative country images associated with emerging economies (Ramachandran & Pant, 2010; Verlegh & Steenkamp, 1999). Smaller firms lack the resources for costly reputation-building activities, and personal networks have proved key to the internationalization of small- and medium-sized firms (SMEs) from emerging economies in the past (Ciravegna, Lopez, & Kundu, 2014; Musteen, Datta, & Butts, 2014), but few people in emerging markets have international personal contacts. We showed that the signaling mechanisms provided by platforms helped to reduce statistical discrimination against providers from lower-income countries (Hypothesis 3). They did not fully eradicate statistical discrimination but had a very substantial effect. While our quantitative analysis was confined to examining discrimination along the low-/high-income axis, qualitative findings suggest that platform signaling was able to overcome country stereotyping more generally. Platform-signaling mechanisms are economical for even the smallest providers and thus give rise to individualization, or the novel phenomenon of one-person microproviders, including especially from emerging economies, serving international clients.

### Future Research on the Global Platform Economy

Our qualitative findings further underline that platform signaling mechanisms are critical to microproviders as they seek foreign clients. Indeed, the platform is perceived not necessarily merely as a passive signaling environment but as an active participant in a triadic provider-platform-client relationship, for example, when the platform recognizes the provider’s “expert” status. But like the MNE or offshoring firm, the platform has its own interests, which in many but not all ways overlap with the interests of individuals seeking to earn a return on their skills. Although microproviders can choose between several global platforms, the quality signals that they accumulate on a platform create a switching cost that grows over time, making it possible for a rent-seeking platform owner to capture a large share of the value. Future research should thus address global platforms’ implications for aspects like dependence, value capture, and working conditions in the same way as it has scrutinized such effects for MNEs and global value chains (Gereffi & Lee, 2016; Graham et al., 2017; Strike, Gao, & Bansal, 2006).

Another phenomenon that warrants scrutiny is taste-based discrimination. It differs from statistical discrimination in that it does not stem from a lack of knowledge but is based on a preference for or animus against a certain group. There are possible indications of taste-based discrimination online: Ghani et al. (2014) find that Indian diasporan buyers on labor platforms prefer India-based providers, and Edelman, Luca, and Svirsky (2017) suggest that taste-based discrimination is prevalent in Airbnb (but cannot rule out statistical discrimination). This suggests various avenues for future research: first, to what extent and in which ways taste-based discrimination is experienced on global platforms and, second, to what extent preferences are “acceptable” and to what extent discriminatory. For example, in our study, English language skills resemble general skills tests in their signaling effectiveness. But language could be a marker of competence or of ethnicity, and future research is needed to tease out effects. Finally, the information provided through platforms can complement or perhaps even diminish the importance of the home country institutional context in reducing discrimination. Especially for providers from emerging economies, this avenue for foreign market success needs further study.

While our study approached the global platform economy from the perspective of microproviders, future research should also address the client perspective. As a new offshoring mode, the global platform economy introduces additional options for the management of global work. While outsourcing and hybrid workforces have been discussed extensively in the human resource management literature, the global platform economy takes this trend to the level of the individual. Implications to both strategic human resources management and everyday human resources practices should be investigated (for emerging research, see Corporaal & Lehdonvirta, 2017).

Our study used a mix of two data sources with very different characteristics that to some extent offset each other’s limitations. Analyses of digital trace data (“big data”) generated results with excellent internal validity, while interview data helped to validate these findings in a social context and generalize them beyond a single platform and across broader types of work. This combination—as well as our strategy for directly juxtaposing quantitative and qualitative findings—offers a useful model that we hope other scholars using big data can use to ensure the robustness of their findings.

Although in this study we focused on labor platforms, our theory of global platforms as institutions is likely to be equally applicable to other factor and product markets, where platforms will have similar implications. For instance, Alibaba has emerged as a significant platform for international trade in intermediate and final products, especially among SMEs and microenterprises. Many “crowdfunding” platforms have emerged to facilitate international flows of capital to and from SMEs, microenterprises, and individual investors. In each case, the global platform economy is addressing hitherto-underserved needs as well as offering a challenge to incumbent institutions, in a process characterized by glocalization, individualization, and the powerful role of platforms as mediators.

## Appendix

### Methodology of Qualitative Component

The data presented in this article were generated as part of a 3-year, multistage, mixed-methods research project investigating the global platform economy. Embracing a sequential research design (Creswell & Clark, 2011; Johnson, Onwuegbuzie, & Turner, 2007), the most comprehensive empirical phase sought to elicit data on the lived experiences of online freelance workers or microproviders by means of in-depth semistructured interviews carried out in face-to-face settings with the providers during 7 months of fieldwork in Southeast Asia and Sub-Saharan Africa taking place between September 2014 and December 2015. The countries represented are all lower- to middle-income countries that are regarded as emerging economies: the Philippines, Malaysia, Vietnam, South Africa, Kenya, and Nigeria.

#### Sampling of microproviders through online platforms

In qualitative research, sampling strategies are not as much concerned with questions of “representativeness” as with questions of conceptual fit (Miles & Huberman, 1994: 29). For interview studies, Morse (2012) highlights the notion that qualitative sampling should be representative of the phenomena rather than of the population. These conceptualizations of sampling guided the participant selection and recruitment process. As an outcome, the first participant inclusion criterion was active membership of any online labor market platform operating in the six countries listed above.

Ultimately, we recruited microproviders who bid for work through the following platforms: Elance, freelancer.com, oDesk, and PeoplePerHour. Many participants sought work through several platforms at once. Microproviders’ experiences captured by the interview data thus also included engagement with additional platforms, such as iWriter.com, guru.com, rev.com, and others.

The second participant selection criterion devised was that the work performed should constitute low-skilled tasks that did not require any formal qualification. The sampling strategy, however, in accordance with contemporary methodological theorizing, was “fluid and emerging throughout [the] research design, from research questions to data analysis” (Beitin, 2012: 243). This fluidity, for example, pertained to work categories included in recruitment search processes facilitated through the various online platforms identified. This was because the task ontologies employed by platforms were nonexhaustive, unclear, and/or inconsistently used by clients and providers alike, as early-stage fieldwork and interviews revealed.

Furthermore, we often found that providers were not specializing in only one category of work, such as data entry, but often performed any type of work they had the skills to do. In addition, microproviders with many years of experience often sought to upskill themselves in order to meet the skills demands of platform clients, allowing them to be considered for the types of jobs they observed were most frequently offered at higher rates. As an outcome, the range of worker experiences generated through the semistructured interviews span from low- to high-skilled tasks. The majority of participants, however, engaged with tasks such as blog/article writing, search engine optimization, data entry, virtual assistant services, transcription, lead generation, and e-mail handling.

Finally, we sought to ensure a rough gender balance among the participants. These criteria were then applied to select and recruit microproviders for interviews.

#### Recruitment of microproviders for interviews

The actual process of recruiting interview participants is a stage of the qualitative research process that remains underreported and undertheorized (Kristensen & Ravn, 2015). Yet, it is “both time-consuming and personally and professionally challenging” (Kristensen & Ravn, 2015: 725), and is a key methodological and validity concern in studies using qualitative interviews (O’Connor & Madge, 2017). As indicated above, in the sampling and selection process, we explicitly harnessed the platforms as tools for recruitment. In particular, the platforms’ search engines were valuable tools for exploring trends and identifying providers in the geographical locations of interest. Furthermore, many platforms offered a wide range of additional information on workers’ characteristics, such as the amount of money earned through the platform, their hourly rates, the number of hours they had billed, their level of English language skills, and importantly, information on when they were last active on the platform.

For interview research relying on recollections of everyday experiences of online work, specific tasks, client relationships, and similar information, the likelihood of generating high-quality of data is higher if the events and experiences in question have happened more recently. The conscious use of the sociotechnical affordances of the platforms as tools for selecting and recruiting participants enabled the representation of a broad “variety of positions in relation to the research topic” (King & Horrocks, 2010: 29).

To invite microproviders to participate in the research interviews, the research team posted tasks on the four online platforms mentioned. The task descriptions provided information about the research project, included questions relating to informed consent, and clarified that participation was voluntary and should not be seen as a job. To further make that point, it was emphasized that no feedback (reputation score) would be left as an outcome of the participation. Expenses for transport would be reimbursed, and participants would receive $6 as a token of gratitude; the latter was not conditional on following through with the interview and would be paid to anyone accepting the invitation. The tasks were “hidden,” meaning that only individuals manually selected as outlined above were able to access the task description and accept the task.

Nonetheless, a key validity concern was ensuring that participants genuinely perceived the interview situation as research participation and their role as that of informant rather than someone performing a paid task. This was achieved by means of building of relationship and trust. A one-off encounter and interaction would inhibit the more genuine building of rapport and relationship between researcher and participant. To facilitate continuity and the building of rapport, we devised a research process and protocol that ensured that participants engaged with the lead interviewing researcher (Hjorth) in various ways prior and subsequent to the interview itself. In most cases, the researcher and the participant had exchanged several rounds of e-mails, platform chat messages, and SMS messages by the time they met face-to-face. Repeated exchanges of e-mails, messages, and artefacts have been found to effectively help build rapport (Deakin & Wakefield, 2014; Seitz, 2016). Artefacts shared by participants included materials such as their curricula vitae, LinkedIn and other professional or personal social media profiles, personal blogs, microfinancing campaigns, and similar. When possible, these were used as prompts during interviews and included as contextual data in the analysis phases.

#### Interview protocol

In total, 107 emerging-economy microproviders were interviewed, in the Philippines (*n* = 12), Malaysia (*n* = 5), Vietnam (*n* = 19), South Africa (*n* = 19), Kenya (*n* = 29), and Nigeria (*n* = 23). All interviews were carried out in English, in some instances with support from a local research assistant/interpreter. Most interviews lasted around 1.5 hr. Informed consent was ensured by means of an information sheet and consent form.

An interview guide was used to provide overall structure to the interviews, resulting in a semistructured interview design. The interview guide covered the participant’s socioeconomic background, educational and employment history, experiences and practices related to working through online labor platforms, family and social networks, engagement with technology, and personal aspirations. Follow-up questions were used to probe topics further, and the majority of the interviews were spent discussing the participant’s experiences and practices related to online labor platforms.

#### Approach to qualitative data analysis

The interviews were recorded and the recordings were transcribed. An initial analysis phase took place in the form of reflection in debriefing meetings among the research team after individual interviews and fieldwork periods. A more formal analysis phase took place after the fieldwork was finished, when the interview transcripts were read and excerpts coded in NVivo using Miles and Huberman’s (1994) notions of first- and second-level coding. The initial coding resulted in hundreds of first-level codes that were partly descriptive (e.g., socioeconomic background, platform preference) and partly theoretical (reflecting literature on global sourcing, economic geography, and labor sociology that informed the overall research project). The initial codes were then merged into higher-level codes in an iterative process.

In this article, the purpose of using interview data was to augment the findings from quantitative analyses, offering convergent validation (triangulation) as well as increasing the “analytical density” (Fielding, 2012) of the research with rich, noisy data, which is not formally generalizable but helps to offset some of the limitations of the quantitative data and provide context for their interpretation (Bryman, 2006; Neff, Tanweer, Fiore-Gartland, & Osburn, 2017). This was achieved through a third analysis phase that consisted of identifying higher-level codes relevant to the questions addressed by the quantitative analyses, examining the first-level codes they encompassed, and reviewing related transcripts for context. This resulted in a narrative that outlines participants’ experiences and practices related to the questions addressed by the quantitative analyses, which is juxtaposed in the article with the quantitative results and finally synthesized with them into a “negotiated account.” The top-level codes used in this analysis are listed in Table A1.

**Table A1:** Top-Level Codes Used in the Analysis

Top-Level Code	Excerpts (Including Lower-Level Codes)
Formal qualifications and educational trajectory	192
Employment history and trajectory	254
Online work history and entry	248
Motivations for doing online work	111
Reputation/feedback systems	244
Perceived influences of geography	259
Discrimination	164
Platform design, policies, and practices	36
